# Artificial intelligence in oncology: chances and pitfalls

**DOI:** 10.1007/s00432-023-04666-6

**Published:** 2023-03-15

**Authors:** Jakob Nikolas Kather

**Affiliations:** Else Kröner Fresenius Zentrum für Digitale Gesundheit and Medizinische Klinik und Poliklinik 1, Universitätsklinikum Carl Gustav Carus, Technische Universität Dresden, Fetscherstr. 74, 01307 Dresden, Germany

**Keywords:** Artificial intelligence, Hematology, Oncology, Large language models

## Abstract

Artificial intelligence (AI) has been available in rudimentary forms for many decades. Early AI programs were successful in niche areas such as chess or handwriting recognition. However, AI methods had little practical impact on the practice of medicine until recently. Beginning around 2012, AI has emerged as an increasingly important tool in healthcare, and AI-based devices are now approved for clinical use. These devices are capable of processing image data, making diagnoses, and predicting biomarkers for solid tumors, among other applications. Despite this progress, the development of AI in medicine is still in its early stages, and there have been exponential technical advancements since 2022, with some AI programs now demonstrating human-level understanding of image and text data. In the past, technical advances have led to new medical applications with a delay of a few years. Therefore, now we might be at the beginning of a new era in which AI will become even more important in clinical practice. It is essential that this transformation is humane and evidence based, and physicians must take a leading role in ensuring this, particularly in hematology and oncology.

## Established AI applications in cancer medicine

Currently, the majority of AI applications in cancer medicine are related to digital image processing in fields such as dermatology, endoscopy, radiology and pathology (Benjamens et al. 2020; Shmatko et al. 2022; Rajpurkar et al. 2022). There is extensive clinical evidence available for AI systems in some of these specialties, such as for colorectal cancer screening with endoscopy (Ngu et al. 2019), lung cancer screening with radiology imaging (Nam 2023) and biomarker prediction from histopathology images (Saillard et al. 2022; Kleppe et al. 2022). In summary, AI for medical image analysis is a mature technology and can be expected to be applied to many more use cases in oncology in the next few years. Yet, even beyond image analysis, machine learning systems have been shown to improve the practice of oncology: one of the largest study in this field included over 20,000 patients with cancer and showed that using a machine learning system to confront oncologists with patient prognosis led to an increase in the number of serious illness conversations (SICs) between doctors and patients with cancer, and also reduce the use of aggressive treatment at the end of life (Manz et al. 2023). This evidence is encouraging because it shows that AI can help to improve quality of life in human-centered medicine. More examples for the use of AI can be found in the consensus statement of the joint Working Group on “Artificial Intelligence in Hematology and Oncology” by the German Society of Hematology and Oncology (DGHO), the German Association for Medical Informatics, Biometry and Epidemiology (GMDS), and the Special Interest Group Digital Health of the German Informatics Society (GI), which is published in the same issue of this journal.

## Recent developments of medical AI in 2022

Since 2022, some of the most striking advances have been in the area of natural language processing (AI). Breakthroughs in large language models (LLMs) have moved this field of AI in the center of public attention. LLMs are AI models which can understand and synthesize text with human-level performance. LLMs can understand, summarize and write scientific articles, understand and write computer code and medical texts, they can converse and make jokes. Google, Microsoft-backed OpenAI and other large technology companies are pushing LLMs towards medical applications, such as information retrieval, summary, chatbot functionalities, among many other potential applications (Singhal et al. 2022). An LLM chatbot with medical expert knowledge is not science fiction anymore. We are conceivably just months away from such an AI system. Thus, ironically, radiology and pathology might not be the medical specialties most affected by AI, but through LLMs, internal medicine including hematology and oncology could be massively influenced and changed by LLMs (Health 2023; Patel and Lam 2023). However, while current LLMs are impressive at first glance, a deeper analysis reveals that they often hallucinate factually incorrect information, not attribute references correctly, and be overconfident in wrong or biased output. Developing safeguards and guaranteeing alignment of LLM chatbots with evidence based and human-centered medicine is a massive endeavor for the next few years which needs to be led by physicians. To enable this, it will be key to support physicians in building AI literacy.

## Building AI literacy

The rapid development of AI requires physicians to stay up to date and comprehend the medical implications of these technologies. Being digitally literate and capable of critically evaluating clinical evidence in the AI era is a fundamental skill which physicians must build and cultivate. All physicians must become aware of AI and comprehend its fundamental principles. In addition, some physicians might choose to delve deeper into AI and actively use it as a tool for research. By enabling the processing of large amounts of unstructured information, AI could fundamentally alter and transform nearly all aspects of contemporary medicine, including preclinical research, drug discovery, clinical trials, and even clinical routine activities, including communication. We are living in exciting times and the world of hematology and oncology as we know it will change in the future (Topol 2019). In these times, it will be crucial to focus on the fundamental principles of modern hematology and oncology, such as the primacy of empirical evidence, critical thinking, shared decision-making, and the importance of having an empathetic individual at the center of healthcare.

## Data Availability

No data was analyzed in this article.
